# Smoking as a product of gene–environment interaction

**DOI:** 10.1080/03009730902833406

**Published:** 2009-04-24

**Authors:** Kent W Nilsson, Lars Oreland, Robert Kronstrand, Jerzy Leppert

**Affiliations:** ^1^Centre for Clinical Research, Uppsala University, Central Hospital721 89 VästeråsSweden; ^2^Department of Neuroscience, Unit of Pharmacology, Uppsala University, BMCBox 593, 751 24 UppsalaSweden; ^3^National Board of Forensic Medicine, Department of Forensic Chemistry, University Hospital581 85 LinköpingSweden

**Keywords:** Adolescents, cotinine, environment, gene, nicotine, serotonin, smoking

## Abstract

A strong hereditary influence on smoking has been demonstrated. As one of the candidate genes in relation to smoking, the serotonin transporter gene (*5-HTTLPR*) has been suggested, however with conflicting results. In recent studies, it has been shown that genotypic and environmental (G*E) factors interact in the shaping of a variety of phenotypic expressions. The objective of the present study was to investigate the interaction between a variation in the *5-HTTLPR* and family environment in relation to smoking habits, nicotine dependence, and nicotine and cotinine levels in hair samples.

A random Swedish adolescent population sample (*n =* 785), from which 200 individuals were stratified regarding behaviour, was genotyped for *5-HTTLPR* and assessed with semi-structured interviews, a questionnaire, and hair analyses of nicotine and cotinine.

The *5-HTTLPR* gene interacted with a poor family environment to predict smoking habits, as well as nicotine and cotinine levels. The risk of being a smoker was increased 13 times for an individual with a combination of the *5-HTTLPR* LS genotype and a poor family environment in comparison with the Homozygous Long-Long (LL) genotype and a good family environment.

## Introduction

Tobacco consumption and smoking are the most important and preventable causes of mortality and morbidity in Western societies. Globally the tobacco consumption is rising because of increased consumption in many low-income countries (1). Smoking behaviour is influenced by both genetic and environmental factors (2–5). The genetic influence on smoking is reported to be approximately 60% (46%–84%) whereas about 20% of the variation comes from shared and 20% from individual environmental influences (5,6). However, among those who develop nicotine dependence, genetic factors appear to account for about 70% while shared environmental influences are close to negligible (6).

Different polymorphisms have been identified in the promoter region of the serotonin transporter, one of which is an insertion/deletion polymorphism in the upstream regulatory region (*5-HTTLPR*) (7). It has been demonstrated that the long (L) and short (S) variants of this 5-HTT gene-linked polymorphic region (5-HTTLPR) have different transcriptional activities. Functionality studies of the 5-HTT gene transcription of *5-HTTLPR* have shown that the short variant is associated with lower expression of 5-HTT and lower 5-HT reuptake activity in lymphoblasts and placental choriocarcinoma (JAR) cells (8,9). The *5-HTTLPR* polymorphism has been related to smoking, with conflicting results, however. In two studies, the long variant was more frequent among smokers (10,11). Gerra et al. 2005 on the other hand, reported an over-representation of the short variant among adolescent smokers. Although some studies failed to find any association (12,13), a meta-analysis found modest effects of *5-HTTLPR* on smoking cessation (14). Additional links between the *5-HTTLPR* gene and smoking can be hypothesized as adolescent smokers experience more severe withdrawal symptoms upon smoking cessation than do adults, even when daily smoking has occurred for only a short period or with low levels of consumption (15,16). Among adolescent rats in comparison with adults, withdrawal was accompanied by a significant and persistent loss of striatal 5-HT synaptic activity. In support of this there was an initial decline in turn-over followed by a reduction in 5-HT content without a compensatory increase in turn-over points, suggesting a potential for serotonin-specific reuptake inhibitors as alternatives to nicotine replacement therapy for smoking cessation in adolescents (17). Another link between the *5-HTTLPR* gene and smoking could be that selective serotonin reuptake inhibitors like fluoxetine antagonize the ability of nicotine to evoke hippocampal noradrenaline release *in vitro* (18).

During recent years, links between serotonergic function and environmental factors have been shown in several reports. Gene–environmental (G*E) models of interaction between environment and *5-HTTLPR* for the risk of periods of depression (19–21) and between environment and the *MAO-A* promoter polymorphism for the risk of antisocial behaviour (22–25) have been reported. Furthermore, a G*E model of smoking initiation (serotonin receptor HTR6, lifetime traumatic experiences, and novelty-seeking), has been suggested (26).

The objectives of the present study were to investigate *5-HTTLPR* gene–environmental interactions in relation to smoking behaviour, nicotine dependence, and levels of nicotine and cotinine in hair samples. Our hypothesis was that *5-HTTLPR* would interact with an unfavourable family environment to predict increased risk for smoking, nicotine dependence, and nicotine and cotinine levels in hair samples.

## Material and methods

### Subjects

All ninth-grade students in primary school (*n =* 2987, mean age 16.0 years) and third-grade students in secondary school (*n =* 2186, mean age 19.2 years) in Västmanland, a medium-sized county of Sweden, comprised the target population for the ‘Survey of Adolescent Life in Vestmanland (SALVe)’. In total, 2611 16-year-olds and 1649 19-year-olds, 87% and 75%, respectively, completed the questionnaire. Depending on risk behaviour reported in the questionnaire, all students were classified with a risk index (based on risk behaviours related to alcohol, narcotics, sex, property offences, and violent offences, please see Nilsson (27) p. 64–65) and divided into four groups accordingly. Randomized samples of 400 students, matched for age, gender, and risk behaviour, were drawn from those students who accepted to take part in further investigations. A total of 81 boys and 119 girls gave blood and hair samples and took part in an interview. There were no differences between the group interviewed and those students responding to the initial questionnaire with regard to smoking habits (29% versus 33%, *P =* 0.300). Three years after the initial interview and the sampling of blood and hair, a follow-up questionnaire on smoking and smoking habits was sent to the participants; 65 boys and 112 girls completed the follow-up study and comprised our study group. The study design was approved by the human ethical committee at Uppsala University.

### 5-HTTLPR gene analysis

DNA was extracted from venous blood and analysed for *5-HTTLPR* polymorphism. Polymerase Chain Reaction (PCR)-based genotyping was performed according to a modified protocol by Collier (8). In order to confirm that the correct regions of the 5-HTT gene were amplified, PCR products representing all genotypes were sequenced using BigDye® Terminator chemistry (Applied Biosystems) and analysed by an automated ABI PRISM^TM^ (Perkin Elmer, Foster city, CA, USA). The DNA fragments were analysed using the Sequencer^TM^ 3.1.1 software.

### Nicotine and cotinine hair analysis

Analysis of nicotine and cotinine in hair was performed as previously described (28). Briefly, approximately 20 mg of hair were incubated in 0.5 mL of methanol:acetonitrile:buffer (10:10:80) at 37°C for 18 hours. A 150-µL aliquot was transferred to an autosampler vial, 10 µL were injected into a liquid chromatography tandem mass spectrometry (LC-MS-MS) system, and the analytes detected in multiple reaction mode.

## Interview structure design

### Definitions of family functioning

The psycho-social variables were measured by the questions, ‘Could you describe your family?’, ‘What is good about your family, your mother/father and siblings?’, ‘What is not so good about your family?’, ‘What was it like in your family when you were seven, … thirteen?’, and ‘Have there ever been any tough or hard periods within your family?’ If the respondent described controversies within the family, these were followed up. The family relations were first coded on a five-point scale from very good (=1) to very poor (=5). The second stage of the coding process comprised the psycho-social variable ‘quality of relations within the family’ (1–2 = good, and 3–5 = poor). Inter-rater reliability (Cohen's κ) of family functioning, assessed by two independent raters who listened to the audiotaped interviews, was 0.7.

For a further description of participants, procedure, and coding, see (27,29,30).

### Smoking and nicotine tolerance/dependence

In the present study, smokers were defined as individuals who smoke daily or occasionally, whereas non-smokers were defined as individuals who do not smoke at all, i.e. had never started or had stopped smoking. A questionnaire on smoking and smoking habits including the modified version of the Fagerstrom Tolerance Questionnaire (FTQ) was used. The findings of the FTQ provide preliminary evidence that the modified FTQ scale is valid and applicable to adolescent smokers (31,32). The FTQ has been proved to be highly reliable (32).

### Statistical analysis

The Mann-Whitney and Kruskal-Wallis non-parametric tests were used to analyse differences in mean rank of the smokers versus non-smokers, nicotine and cotinine levels and gene, environment and gene–environment subgroups. To analyse the relationship between the results of the Fagerstrom Tolerance Questionnaire (FTQ), nicotine and cotinine, we used Spearman's *r*. The gene–environment interaction model of smoking, nicotine, and cotinine (below and above median) was analysed with logistic regression.

## Results

### Descriptive results

Among the participants, 80 (45%) were smokers (daily or occasional), of which 29 were boys and 51 were girls, with equal distribution of smokers in both sexes. However, 21 boys (32%) and 26 girls (23%) were occasional smokers. A total of 125 adolescents (71%) reported good quality of relations, comprising 45 (69%) boys and 80 (71%) girls. The frequencies of the different genotypes of the *5-HTTLPR* gene were as follows: homozygosity for the short allele variant (SS) = 38 (21.5%), heterozygosity (LS) = 84 (47.5%), and homozygosity for the long variant (LL) = 55 (31%). Among the boys, the corresponding figures were SS = 13 (20%), LS = 27 (42%), and LL = 25 (38%); and among the girls SS = 25 (22%), LS = 57 (51%), and LL = 30 (27%). There were no differences according to the Hardy-Weinberg equilibrium (*P =* 0.451; boys, *P =* 0.407; girls, *P =* 0.477). The Fagerstrom Tolerance Index was significantly correlated to both nicotine (*r =* 0.511, *P <* 0.001) and cotinine levels (*r =* 0.363, *P <* 0.001) in hair samples. The relationship between nicotine and cotinine was *r =* 0.616, *P <* 0.001.

### *5-HTTLPR* and family relations in association with smoking

The proportion of smokers was higher in adolescents from families with poor relations compared to those with good relations, 70% versus 35% (*P <* 0.001), and there was a significantly higher proportion of smokers among heterozygous *5-HTTLPR* (LS) than homozygous individuals (SS = 25%, LS = 70%, and LL = 33%, *P <* 0.001). The same pattern was also found among boys and girls analysed separately.

Adolescents from families with poor relations scored higher in the Fagerstrom Tolerance Index (*P <* 0.001) and had higher nicotine and cotinine levels in hair samples (*P =* 0.003, and *P =* 0.008, respectively). *5-HTTLPR* heterozygous individuals also scored higher in the Fagerstrom Tolerance Index (*P <* 0.001), but a similar association with nicotine and cotinine levels in hair samples was not found. However, when considering gene–environment subgroups there were differences in the Fagerstrom Tolerance Index (*P <* 0.001), nicotine levels (*P =* 0.033), and cotinine levels (*P =* 0.008).

**Figure 1.**
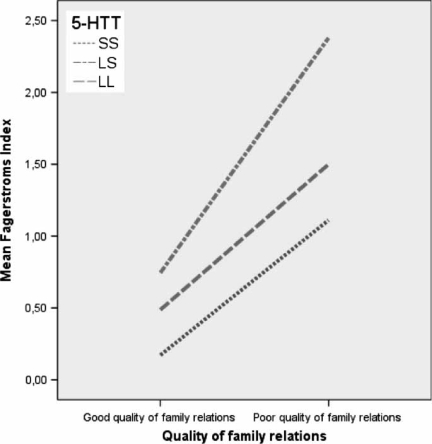
A line-chart of the relationship between the serotonin transporter gene and the quality of family relations in association with the Fagerstrom Nicotine Dependence Index. Homozygous for the long allele (LL), Heterozygous (LS), Homozygous for the short allele (SS).

The gene–environment interaction in relation to the Fagerstrom Tolerance Index is shown in Figure 1. As a group, individuals with poor family relations showed higher tolerance scores, regardless of the *5-HTTLPR* variant. When at environmental risk, *5-HTTLPR* heterozygous individuals showed higher Fagerstrom Tolerance Indexes than both groups of homozygotes.

Using logistic regression (Table I), both the quality of family relations and the *5-HTTLPR* genotype were independently and significantly related to smoking in separate models (models 1 and 2), and adjusted for each other in the same model (model 3). A heterozygous individual had about three times elevated odds ratio (OR) of being a smoker. Poor family relations resulted in somewhat increased odds. However, individuals carrying the *5-HTTLPR* LS variant and reporting poor family relations had elevated odds of 13 times (95% CI for OR, 4.9–42.8) for being a smoker compared to LL individuals with good family relations. Moreover, the explained variance of the G*E interaction was over 23%.

**Table I.** Logistic regression models of 1) *5-HTTLPR* genotype, 2) quality of family relations, 3) model adjusted for 5-HTTLPR and quality of family relations, and 4) gene and environment interaction in relation to smoking among adolescents.

	OR	95.0% CI for OR	*P*
1) *5-HTTLPR*			
LL (ref)			
LS	3.34	1.63–6.83	0.001
SS	0.68	0.28–1.70	0.41
Nagelkerke *R*^*2*^=0.141			
2) Quality of family relations			
Good quality of family relations (ref)			
Poor quality of family relations	4.26	2.13–8.50	<0.001
Nagelkerke *R*^*2*^=0.13			
3) Adjusted model			
LL (ref)			
LS	3.23	1.56–6.99	0.001
SS	0.73	0.28–1.91	0.53
Good quality of family relations (ref)			
Poor quality of family relations	4.09	1.97–8.49	<0.001
Nagelkerke *R*^*2*^=0.24			
4) Final interaction model			
LL–Good relation (ref)			
LS–Good relation	2.83	1.18–6.75	0.019
SS–Good relation	0.57	0.17–1.86	0.35
LL–Poor relation	2.73	0.78–9.57	0.12
LS–Poor relation	13.04	4.90–42.84	<0.001
SS–Poor relation	3.41	0.77–15.05	0.11
Nagelkerke *R*^*2*^=0.24			

Homozygous for the long allele (LL), Heterozygous (LS), Homozygous for the short allele (SS).

**Figure 2.**
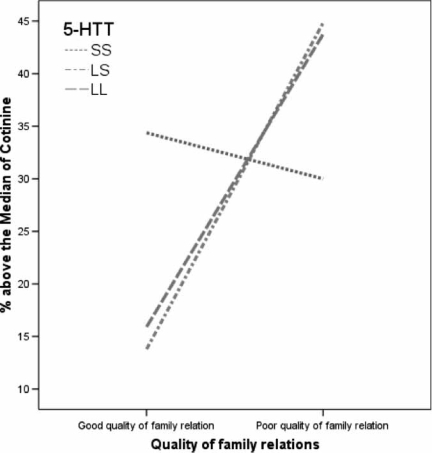
A line-chart of the relationship between the serotonin transporter gene and the quality of family relations in association with proportion of individuals with cotinine levels above the median in the total sample. Homozygous for the long allele (LL), Heterozygous (LS), Homozygous for the short allele (SS).

Likewise, there was a significant gene–environment interaction in relation to nicotine and cotinine levels (below versus above the median) tested with logistic regression (Table II). The explained variance (Nagelkerke *R*^*2*^) using the nicotine and the cotinine levels model was 7% and 12%, respectively, with elevated odds (OR 3.2–4.3) comparable with that in the smoking model (Table I) for LS individuals from a family with poor relations. The gene–environment interaction in relation to cotinine levels in the analysed hair samples is illustrated in Figure 2. It can be observed that the group of *5-HTTLPR* SS from families with good quality of relations has a higher proportion with cotinine levels higher than the median compared to LL individuals with a similar family background (*P =* 0.07 in the regression model). However, among the *5-HTTLPR* LL and LS individuals there was a markedly higher proportion with cotinine levels above the median, when poor family relations were considered (*P =* 0.03 and 0.009, respectively).

**Table II.** Logistic regression models of gene and environment interaction in relation to nicotine and cotinine among adolescents.

	OR	95.0% CI for OR	*P*
Nicotine: Final interaction model			
LL—Good relation (ref)			
LS—Good relation	1.16	0.54–2.59	0.71
SS—Good relation	1.12	0.45–2.82	0.80
LL—Poor relation	3.18	0.94–10.72	0.06
LS—Poor relation	3.21	1.19–8.64	0.02
SS—Poor relation	2.17	0.53–8.79	0.28
Nagelkerke *R*^*2*^=0.07			
Cotinine: Final interaction model			
LL—Good relation (ref)			
LS—Good relation	0.85	0.28–2.54	0.76
SS—Good relation	2.77	0.93–8.22	0.07
LL—Poor relation	4.11	1.15–14.73	0.03
LS—Poor relation	4.30	1.44–12.77	0.009
SS—Poor relation	2.26	0.47–10.95	0.31
Nagelkerke *R*^*2*^=0.12			

Homozygous for the long allele (LL), Heterozygous (LS), Homozygous for the short allele (SS).

## Discussion

The present study shows an association between *5-HTTLPR* and smoking. This association was considerably amplified by interaction with the environment, where *5-HTTLPR* heterozygous (LS) individuals with poor family relations had the highest risk of smoking with an OR of 13 compared to individuals homozygous for the long allele (LL) with good family relations. A similar, although with lower OR, gene–environment interaction pattern was found in relation to levels of nicotine and cotinine contents in hair samples. However, snuff is a commonly used tobacco product in Sweden. The nicotine and cotinine levels in the hair samples might have been influenced by snuff-taking. That could be a reason that lower OR was found for the gene–environment interaction in relation to levels of nicotine and cotinine contents in hair samples.

There was a relatively high proportion of smokers in our study (45%), which included daily and occasional smokers. The proportion of daily smokers (12% among boys and 22% among girls) did not, however, diverge from that expected according to previous findings in Swedish adolescent populations (33).

In recent studies on humans, acute tryptophan depletion heightened the attentional salience of cigarette-related cues, perhaps by triggering reward and motivational mechanisms underlying nicotine dependence (34). The present study proposes a G*E model of smoking habits involving a functional variation of a gene (5-HT), which has direct implications on serotonergic neurotransmission (35).

Studies on non-human primates with an orthologous variation in the 5-HTT promoter region (*rh5-HTTLPR*) have shown that childhood maternal deprivation interacts with the presence of the S allele of *rh5-HTTLPR* to predict an increased limbic-hypothalamic-pituitary-adrenal axis (HPA) activity (36,37). These animals also had lower levels of the serotonin metabolite 5HIAA in the cerebrospinal fluid (CSF), indicating a lower serotonin turn-over (38). Therefore, also humans with low serotonergic capacity might have higher arousal, which in an insecure family environment might predispose for nicotine use as a substance to control arousal.

Two explanations of gene–environment interaction are often proposed. The first explanation implies that internal and external stimuli, hormones, stress, learning, and social interaction alter the binding of the transcriptional regulators through epigenetic mechanisms (39,40). Recent studies have shown that *5-HTTLPR* polymorphisms have significant effects on 5-HTT mRNA expression in lymphoblast cell lines and that the average methylation was higher among short alleles with trends for linearity with heterozygotes as an intermediate (41,42). These findings may indicate that basic gene effects on the transcriptional level of mRNA expression have to be reconsidered in favour of different environment-induced methylation processes according to individual genotypes.

The other explanation implies that the template function and the transcriptional functions are stable and that an individual's specific expression of, for example, serotonin pathways therefore is stable. Thus, a person with the short allelic variant should have a lower expression of 5-HTT and lower 5-HT reuptake activity (8). Such stable expression levels would then interact with the environment, and a person with a weak serotonergic system would be at a higher risk of negative experiences in a stressful environment.

Polymorphisms of the *5-HTTLPR* gene have been associated with smoking in several studies, although with conflicting results. Such relationships have been found regarding the long variant (10,11) and the short variant (43). In other cases, no relationship could be demonstrated (12,13). In the present study, *5-HTTLPR* heterozygous individuals had an increased risk of smoking in comparison with either of the homozygous genotypes. Such a heterosis effect has been found in up to 50% of all gene association studies (44–46). At a molecular level, such a heterosis effect is not to be expected. At a molecular level, such a heterosis effect is not to be expected and different explanations for the phenomenon have been proposed. The first explanation is based on an inverted U-shaped response curve in which too little or too much gene expression is unfavourable (46,47). The second explanation suggests greater plasticity in heterozygous individuals, e.g. in their responses to stress, due to a broader range of gene expression when compared to individuals homozygous for either the short or the long allele (46). In the case of *5-HTTLPR*, LS individuals have been shown to have fewer serotonin transporter binding sites in several mid-brain regions (48), higher proportions of females with seasonal affective disorder and premenstrual dysphoric disorder (49), elevated triglyceride and cholesterol levels, angina and heart disease (50). Therefore, a reverse heterosis functionality is plausible; the adolescent homozygous individuals are more ‘vulnerable’ and perhaps also more sensitive to environmental exposure. Consequently, LS individuals might be more susceptible to and more affected by artificial (nicotine) elevations of striatal 5-HT synaptic activity (17).

The major limitation of the present study is the low number of subjects. It has often been proposed that a lack of significant results in genotype–phenotype studies is due to small sample sizes, and that small genetic effects need large sample sizes to be detected (51). Explanations of why some gene–environment studies of small samples show significant results, whereas other large studies fail to do so may be sample age and sex composition, and/or assessment methods of the environment and phenotypic expression (52). However, the present study demonstrates that statistically significant results on genetic contributions to smoking behaviour, measured by self-reports, can be observed if models are adjusted for environmental factors. Moreover, biological markers of smoking, such as nicotine and especially cotinine, are definitely more stable measures than self-reports of smoking and give solid additional support for the results of the present study.

Another important limitation of our study is that it relies on self-reports on family relations. Such reports regarding potentially ambiguous circumstances that may be affected by subjective retrospective interpretations and reconstructions, such as whether a person has experienced poor parenting, should be interpreted with caution (53–55).

A third limitation is that our data are based on association, which means that conclusions regarding the directions of cause and effect must be considered tentative. Thus, even though we found that self-reported poor family relations may predict smoking behaviour, it may be objected that what we have actually measured might be a tendency of an individual to experience or report his/her family as more negative in terms of its relations. Such tendency might be that an adolescent who smokes has parents who strongly disapprove of the life-style and smoking habits of their offspring, and consequently quarrels or punishments make the adolescent report poor family relations.

The *5-HTTLPR* gene has repeatedly been related to anxiety (56) and to depression in G*E models (19–21). People with current or past depression are more likely to have been smokers at some point in their lives (57). This could be a major confounder in the present study. If there is a causal relation between *5-HTTLPR*, poor family relations, and depression, then depression could act as a mediator in relation to smoking. However, the explained variance in G*E–depression studies seldom reaches above 5% (19–21), whereas this study shows a substantially higher explained variance. One intriguing possibility could therefore be that the G*E interaction firstly shows re lation to smoking, in which nicotine and/or other toxic compounds modulate the serotonergic synaptic activity in some individuals, leading to depression (58).

The results of the present study suggest a heterosis effect of *5-HTTLPR* in the same way as previously reported in relation to high alcohol consumption (29). However, both reports are based upon the same study population. Further G*E studies in relation to smoking, nicotine dependence, and nicotine and cotinine compounds in hair samples are therefore needed before any confident conclusions can be drawn.
